# Nationwide clinico-epidemiological treatment analysis of adult patients with tumors of cerebellopontine angle and internal acoustic meatus in Poland during 2011–2020

**DOI:** 10.1186/s12889-023-16551-5

**Published:** 2023-09-06

**Authors:** Michał Żurek, Tomasz Wojciechowski, Kazimierz Niemczyk

**Affiliations:** 1 Department of Otorhinolaryngology, Head and Neck Surgery, Medical University of Warsaw, 1a Banacha St., 02097 Warsaw, Poland; 2 Doctoral School, Medical University of Warsaw, 61 Zwirki and Wigury Str, 02091 Warsaw, Poland; 3 Department of Analyses and Strategies, Ministry of Health, 15 Miodowa Str, 00952 Warsaw, Poland

**Keywords:** Cerebellopontine angle tumor, Vestibular schwannoma, Radiotherapy, Microsurgery, Acoustic neuroma

## Abstract

**Objective:**

The aim of this study is to report the epidemiologic characteristics of tumors of the cerebellopontine angle (CPAT) and internal acoustic meatus in adult Polish population throughout the second decade of XXI century and to analyze their treatment.

**Material and methods:**

A retrospective analysis of patients with cerebellopontine angle (CPA) and internal acoustic meatus tumors diagnosed in Poland in 2011–2020 was performed. Data recorded in the National Health Fund (NHF) database were analyzed. International Classification of Diseases codes (ICD-9 and ICD-10) were used to identify study group patients and treatment procedures.

**Results:**

From 2011 to 2020 6,173 Polish adult patients were diagnosed with cerebellopontine angle and internal acoustic meatus tumors. The average incidence in Poland is 1.99 per 100,000 residents/year. It mostly affects women (61.64%), and the average age of patients is 53.78 years. The incidence has steadily increased over the past decade. Treatment has changed significantly over the years, with a definite increase in the number of patients treated with radiotherapy (from 0.54 to 19.34%), and a decrease in surgical therapies (from 41.67 to 6.8%). The most common symptoms were vertigo and/or dizziness (43.48%) and sensorineural hearing loss (39.58%). 4.65% of patients suffered from sudden deafness, in this group of patients the risk of CPAT detection was the highest (6.25 / 1000 patients).

**Conclusions:**

The total incidence of CPAT and demographic characteristics of patients were comparable to other studies. Our study demonstrated the increased number of patients are being treated with radiotherapy and fewer with microsurgery. Sudden sensorineural hearing loss (SSNHL) is an uncommon manifestation of CPAT but proper diagnosis should be undertaken because the risk of diagnosis such tumors is greater in this group.

**Supplementary Information:**

The online version contains supplementary material available at 10.1186/s12889-023-16551-5.

## Introduction

Cerebellopontine angle (CPA) is an anatomical space of posterior cranial fossa bounded by tentorium superiorly, pons and cerebellum posteriorly and temporal bone anterolaterally. Facial and vestibulocochlear nerves run through the CPA from brainstem to internal acoustic meatus (IAM). Anterior inferior cerebellar artery pass closely to these cranial nerves forming a loop that may enter IAM along with them. Due to its deep location and complex topography CPA mass may present with variety of symptoms such hearing loss, tinnitus, dizziness/vertigo or facial paresis.

The most common mass encountered in CPA is vestibular schwannoma (VS). It represents up to 85–90% of tumors in this area followed by meningioma and schwannomas of other cranial nerves (VII and V), so-called non-acoustic CPATs [1, 2]. It is classically described that VS originate from transition zone between the glial and Schwann cells but there are studies that also suggest that it may develop at any point of the nerve, even in vestibular (Scarpa) ganglion where great number of embryonic stem cells and precursors of Schwann cells may be found [3, 4].

Several stage grading systems have been proposed throughout the years depening on the tumor size and its exact location. Commonly used scales include Sterkers, House, Koos and Samii classifications [5]. Currently, the most commonly utilized is Koos system as it has been proven to be reliable method for CPA and IAM tumors classification [6]. As the most common place of origin topographically is the inferior vestibular division of vestibulocochlear nerve in IAM, the mass becomes cerebellopontine angle tumor (CPAT) only if it reaches Koos grade II and higher [1, 6].

The reported incidence of VS in the United States is approximately 1 per 100 000 person-years but it may vary in different ethnic groups from 0.36 per 100 000 person-years in African Americans through Asian Pacific Islanders (1.37 per 100,000 person-years) to the highest reported in Taiwan (2.66 per 100,000 person-years) [7, 8].

As VS is the most common CPAT, unilateral sensorineural hearing loss and tinnitus are the typical signs and symptoms occuring in 95% and more than 60% respectively [9]. Vestibular symptoms and progressive imbalance or dizziness may also be reported by the patient as well as facial paresis, especially in non-acoustic CPATs [2].

Management options for CPATs include treatment, i.e. microsurgical removal or radiation therapy (mainly stereotactic radiotherapy) and “wait and scan” strategy [10]. In Poland, only a few departments of neurosurgery and otorhinolaryngology treat CPATs via retrosigmoid, middle fossa and translabyrinthine approaches. Lately, radiosurgery has also become an option for Polish patients. All these procedures are performed as inpatient treatment. Increased availability of diagnostic imaging has allowed to safely implement “wait and scan” strategy. Until now, there has been no studies reporting the exact amount of tumors diagnosed, managed and treated in Poland.

The aim of this study is to report the epidemiologic characteristics of cerebellopontine angle and internal acoustic meatus tumors in adult Polish population throughout the second decade of XXI century, and to analyze the treatment applied to these lesions according to the database from National Health Fund (NHF).

## Materials and methods

The study is a retrospective and nationwide survey. The data comes from NHF medical database [11] that includes records of all medical data from public and private hospitals in Poland financed from public sources. The diagnoses were coded according to the International Classification of Diseases, 10th Revision (ICD-10), and all procedures performed were coded using the International Classification of Diseases, 9th Revision (ICD-9). NHF database complies also demographical factors like gender, age and place of residence. The data of Polish population were obtained from Statistics Poland [12].

The study group was defined as adult (> 18 years old) patients hospitalized in neurosurgical or otorhinolaryngological departments between January 1, 2011 and December 31, 2020. Diagnoses were identified using ICD-10 code D33.3. Patients who had undergone craniotomies and excision surgeries for brain lesions (ICD-9 codes 01 with extensions) not involving CPA were excluded from the study group. This approach allows to eliminate other types of cranial nerve tumors with the same ICD-10 code treated with surgery. In case of surgical treatment, only those with a confirmed diagnosis of VS were considered. On the contrary, all non-surgical lesions were included notwithstanding the histopathological nature, since this was not achievable in most cases.

The ICD-9 codes 04.011 and 04.012 were used to identify surgical resection of CPATs with confirmed diagnosis of VS. Stereotactic radiosurgery (gamma knife) was identified with ICD-9 codes 92.27 and 92.3 with extensions. Other codes 92.2 with extensions were used to identify other radiotherapies. The symptoms of patients defined using ICD-10 codes presented below:

- sudden hearing loss—H91.2
- sensorineural hearing loss—H90.3, H90.4, H90.5, H90.6, H90.7, H90.8
- tinnitus—H93.1
- headache –R51
- dizziness – R42 or vertigo—H81 with extensions

The descriptive statistics of the CPATs incidence during 2011–2020 in Poland were performed. The incidence of CPATs was stratified by age group and presented for each year separately with corresponding population data. The demographic characteristics were also presented. Statistical analyses included treatment methods and trends were analyzed using proportion tests. *P*-values < 0.05 were considered statistically significant. Symptoms were screened in the year of diagnosis and minimum of one year earlier and the most common were analyzed. R statistical software V. 3.6.2 was used for all analyses.

The Polish Ministry of Health, which is entitled by the Law of Republic of Poland to process the data of the national database of hospitalization, approved the study protocol. In that case we did not need to obtain local Ethics Committee approval; the study adhered to the tenets of the Declaration of Helsinki for research involving human subjects. The study design was a retrospective and nationwide survey which was described in detail in our previous papers [13–16]. The data of all adult patients who were diagnosed with vestibular schwannoma between 2011 and 2020 were extracted from the national database of hospitalization. This database is maintained by the National Health Fund (NHF) and records all medical procedures in public and private hospitals in Poland financed from public sources.

## Results

The analysis included 6,173 adult patients with CPATs hospitalized between 2011 and 2020. The average incidence was 19.87 per million citizens and varied from 6.41 to 35.07 depending on age group. The highest incidence was observed in the 60–69 age group. A steady increase in incidence was observed between 2012 and 2019 (from 11.71 to 29.45 per million residents). The average annual increase rate in incidence amounts to 9.63%. Detailed data are presented in Table 1.

**Table 1:** Incidence of CPATs among Polish adults from 2011 to 2020 by age group

	2011	2012	2013	2014	2015	2016	2017	2018	2019	2020	All
No. age 19–29 years (in thousands)	6,517	6,337	6,115	5,887	5,667	5,469	5,280	5,091	4,917	4,738	56,021
No. of CPA tumours	40	27	22	30	18	39	42	56	48	37	359
Incidence/1,000,000 person-yrs	6.14	4.26	3.6	5.1	3.18	7.13	7.95	11	9.76	7.81	6.41
No. age 30–39 years (in thousands)	6,005	6,123	6239	6,314	6,348	6,330	6,290	6,234	6,145	6,045	62,077
No. of CPA tumours	41	57	47	53	68	86	86	91	104	90	723
Incidence/1,000,000 person-yrs	6.83	9.31	7.53	8.39	10.71	13.58	13.67	14.6	16.92	14.89	11.65
No. age 40–49 years (in thousands)	4,822	4,838	4,879	4,956	5,064	5,202	5,341	5,481	5,632	5,768	51,987
No. of CPA tumours	53	50	41	69	103	106	109	141	160	142	974
Incidence/1,000,000 person-yrs	10.99	10.33	8.4	13.92	20.34	20.38	20.41	25.72	28.41	24.62	18.74
No. age 50–59 years (in thousands)	5,765	5,656	5,536	5,406	5,245	5,089	4,928	4,783	4,669	4,605	51,686
No. of CPA tumours	100	91	107	109	142	148	183	172	179	167	1398
Incidence/1,000,000 person-yrs	17.34	16.09	19.33	20.16	27.07	29.08	37.13	35.96	38.33	36.26	27.05
No. age 60–69 years (in thousands)	3,931	4,171	4,409	4,642	4,888	5,024	5,127	5,189	5,219	5,185	47,789
No. of CPA tumours	95	92	108	105	135	203	199	236	259	244	1676
Incidence/1,000,000 person-yrs	24.17	22.06	24.49	22.62	27.62	40.4	38.81	45.48	49.62	47.05	35.07
No. age ≥ 70 years (in thousands)	3,865	3,874	3,883	3,905	3,914	4,030	4,166	4,319	4,484	4,614	41,057
No. of CPA tumours	43	46	59	60	90	128	125	154	165	173	1043
Incidence/1,000,000 person-yrs	11.12	11.87	15.19	15.37	22.99	31.76	30	35.65	36.79	37.49	25.40
No. of all (in thousands)	30,907	31,001	31,064	31,111	31,128	31,146	31,133	31,099	31,068	30,957	310,618
No. of CPA tumours	372	363	384	426	556	710	744	850	915	853	6,173
Incidence/1,000,000 person-yrs	12.04	11.71	12.36	13.69	17.86	22.8	23.9	27.33	29.45	27.55	19.87

**Fig. 1:**
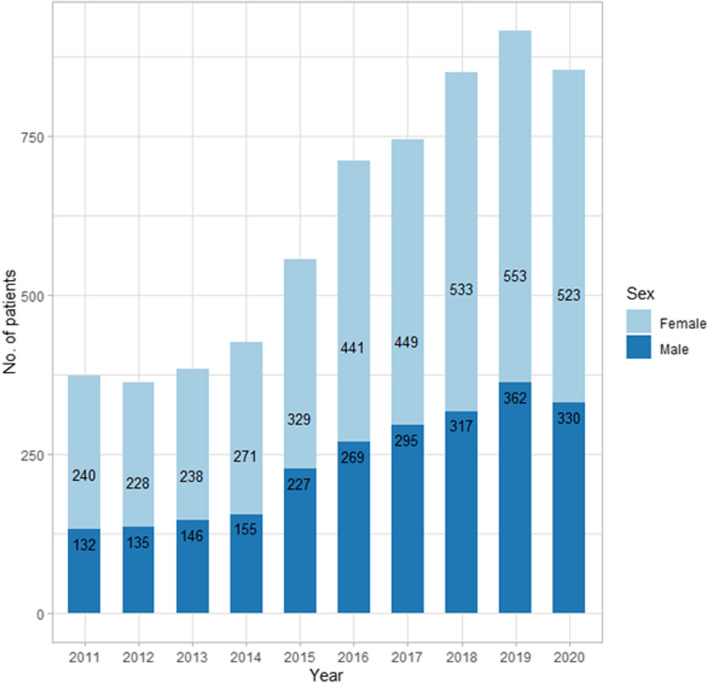
Number of patients with CPATs from 2011 to 2020 in Poland stratified by gender

The average age of patients was 53.78 ± 14.1 years. 61.46% of patients were women, and 70.73% of patients were urban residents. There was no clear trend in the average age, gender distribution or place of residence of patients during the study period. Patient demographics are presented in Table 2 and Fig. 1.

**Table 2:** Demographic characteristics of patients with CPATs among Polish adults from 2011 to 2020

	2011	2012	2013	2014	2015	2016	2017	2018	2019	2020	All
Age mean ± SD	51.92 ± 14.59	53.27 ± 12.39	58.28 ± 12.85	50.75 ± 13.8	53.78 ± 14.25	54.51 ± 14.22	53.96 ± 15.37	52.74 ± 15.27	53.33 ± 14.99	55.27 ± 13.22	53.78 ± 14.1
Women (n,%)	240	228	238	271	329	441	449	533	553	523	3.805
	64.52	62.81	61.98	63.62	59.17	62.11	60.35	62.71	60.44	61.31	61.64
Men (n,%)	132	135	146	155	227	269	295	317	362	330	2.368
	35.48	37.19	38.02	36.38	40.83	37.89	39.65	37.29	39.56	38.69	38.36
Urban residence (n,%)	259	268	279	305	397	509	517	600	648	584	4.366
	69.62	73.83	72.66	71.60	71.40	71.69	69.49	70.59	70.82	68.46	70.73
Rural Residence (n,%)	113	95	105	121	159	201	227	250	267	269	1.807
	30.38	26.17	27.34	28.40	28.60	28.31	30.51	29.41	29.18	31.54	29.27

**Fig. 2:**
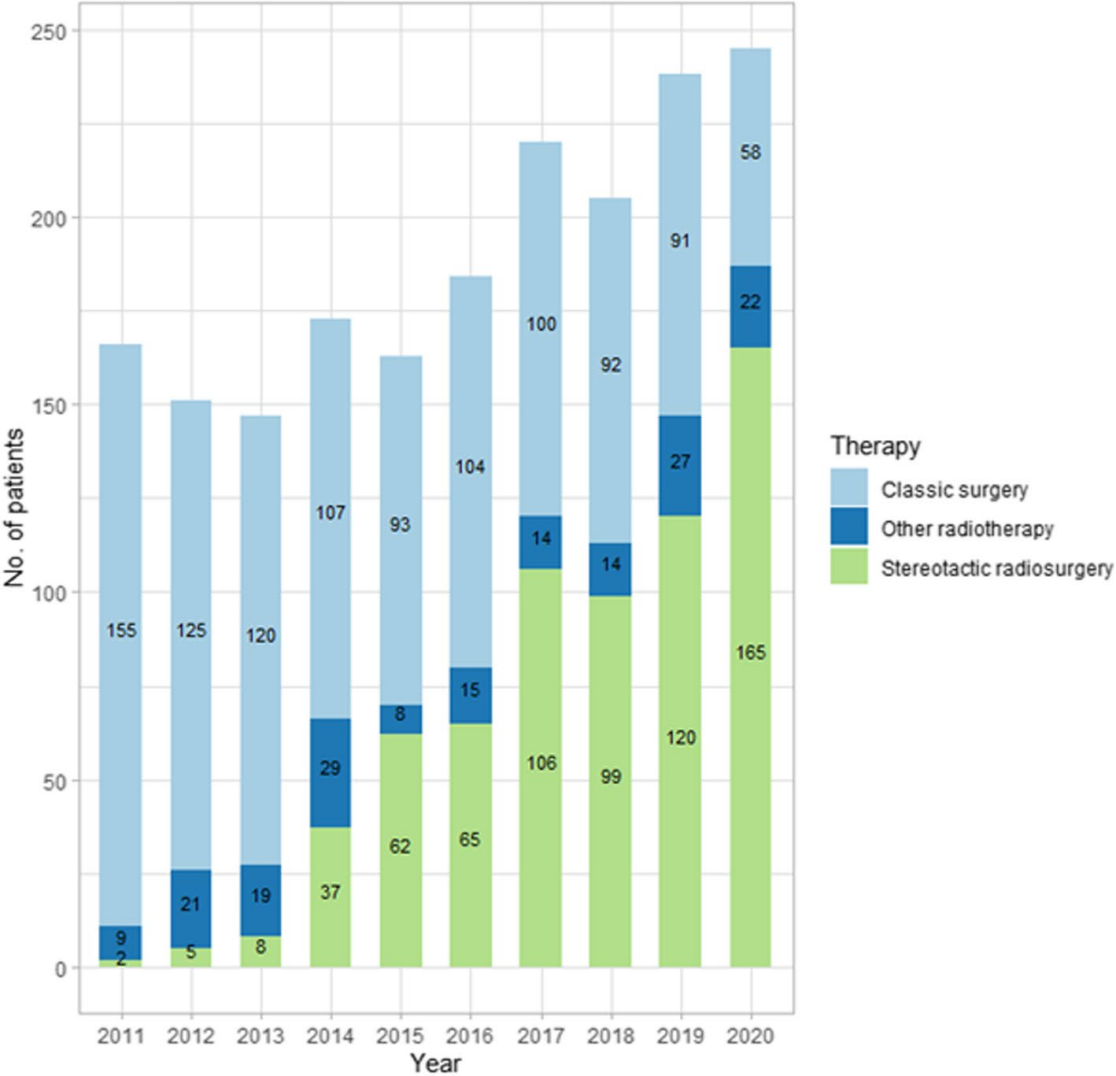
Number of therapies of patients with CPATs from 2011 to 2020 in Poland

During the 10 years analyzed, the treatment methods of patients with CPATs has changed considerably. In the initial period, patients were most often treated surgically, and the percentage of patients has steadily decreased from 41.67% to 6.8%. The opposite trend applied to treatment with stereotactic radiosurgery (mostly gamma knife)—an increase was observed from 0.54% in 2011 to 19.34% in 2020. The number of patients treated with radiotherapy also varied, but without a clear trend (from 1.44% to 5.79%). Patients for whom the use of the above-mentioned treatments was not identified were classified in the "wait and scan" group. This group included between 55.38% and 75.88% of patients. Changes in the percentage of treatment modalities during the study period were statistically significant (for all *p*-value < 0.001). Detailed treatment information are presented in Table 3 and Fig. 2.

**Table 3:** Treatment methods of CPATs among Polish adults from 2011 to 2020

	2011	2012	2013	2014	2015	2016	2017	2018	2019	2020	All	p-value
Surgery (n,%)	155	125	120	107	93	104	100	92	91	58	1,045	< 0.001
	41.67	34.44	31.25	25.12	16.73	14.65	13.44	10.82	9.95	6.80	16.93	
Stereotactic radiosurgery (gamma knife) (n,%)	2	5	8	37	62	65	106	99	120	165	669	< 0.001
	0.54	1.38	2.08	8.69	11.15	9.15	14.25	11.65	13.11	19.34	10.84	
Other radiotherapy (n,%)	9	21	19	29	8	15	14	14	27	22	178	< 0.001
	2.42	5.79	4.95	6.81	1.44	2.11	1.88	1.65	2.95	2.58	2.88	
Wait and scan therapy (n, %)	206	212	237	253	393	526	524	645	677	608	4,281	< 0.001
	55.38	58.4	61.72	59.39	70.68	74.08	70.43	75.88	73.99	71.28	69.39

The symptomatology of CPATs is complex. Sudden sensorineural hearing loss, sensorineural hearing loss, tinnitus, headache, dizziness and vertigo were selected for analysis. The most common symptoms of patients with CPATs were vertigo and/or dizziness (43.48%) and sensorineural hearing loss (39.58%). Less frequently, patients reported headaches (27.7%) and tinnitus (23.29%). Sudden sensorineural hearing loss was the least frequently reported symptom among the selected factors. Only 4.65% of patients with CPAT diagnosed were observed to have such a condition. On the contrary, CPATs was most commonly diagnosed among SSNHL group; on average, 625 out of 100,000 patients with SSNHL were diagnosed with CPAT. CPATs were diagnosed less frequently in patients with tinnitus (141/100,000) and sensorineural hearing loss (124/100,000). There were no obvious trends in the symptomatology of CPATs during the study period. Detailed results are presented in Table 4.

**Table 4:** Clinical characteristics of CPATs among Polish adults from 2011 to 2020

	2011	2012	2013	2014	2015	2016	2017	2018	2019	2020	All
No. of patients with sudden hearing loss (in thousands)	2.6	3.3	3.5	4	4.4	5.1	5.8	6.1	6.3	5	45.9
No. of patients with sudden hearing loss and CPA tumors	12	16	14	18	31	31	39	35	47	44	287
% of all patients with CPA tumors and sudden hearing loss	3.23	4.41	3.65	4.23	5.58	4.37	5.24	4.12	5.14	5.16	4.65
Incidence among patients with sudden hearing loss ( / 100,000 person-yrs)	461.54	484.85	400.00	450.00	704.55	607.84	672.41	573.77	746.03	880.00	625.27
No. of patients with sensorineural hearing loss (in thousands)	110.6	152.2	180.7	199.3	219.3	228.6	231	230	239.1	183.1	1,973.8
No. of patients with sensorineural hearing loss and CPA tumors	114	142	169	180	247	298	319	333	337	304	2443
% of all patients with CPA tumors and sensorineural hearing loss	30.65	39.12	44.01	42.25	44.42	41.97	42.88	39.18	36.83	35.64	39.58
Incidence among patients with sensorineural hearing loss ( / 100,000 person-yrs)	103.07	93.30	93.53	90.32	112.63	130.36	138.10	144.78	140.95	166.03	123.77
No. of patients with tinnitus (in thousands)	49.1	65.9	80.6	92	103.5	112.2	126.5	131.5	139.1	117.8	1,018.1
No. of patients with tinnitus and CPA tumors	68	73	84	100	125	165	192	198	211	222	1,438
% of all patients with CPA tumors and tinnitus	18.28	20.11	21.88	23.47	22.48	23.24	25.81	23.29	23.06	26.03	23.29
Incidence among patients with tinnitus ( / 100,000 person-yrs)	138.49	110.77	104.22	108.70	120.77	147.06	151.78	150.57	151.69	188.46	141.24
No. of patients with headache (in thousands)	373.5	404.5	436.3	482.8	483.3	483.7	490.6	490.2	505.1	371.7	4,521.5
No. of patients with headache and CPA tumors	100	109	111	106	161	209	211	208	247	248	1,710
% of all patients with CPA tumors and headache	26.88	30.03	28.91	24.88	28.96	29.44	28.36	24.47	26.99	29.07	27.70
Incidence among patients with headache ( / 100,000 person-yrs)	26.77	26.95	25.44	21.96	33.31	43.21	43.01	42.43	48.90	66.72	37.82
No. of patients with vertigo (in thousands)	359.8	407.6	453.7	507.6	534.7	569.4	592.4	607	640	526.5	5,198.8
No. of patients with vertigo and CPA tumors	148	142	162	200	237	306	334	360	406	389	2,684
% of all patients with CPA tumors and vertigo	39.78	39.12	42.19	46.95	42.63	43.10	44.89	42.35	44.37	45.60	43.48
Incidence among patients with vertigo ( / 100,000 person-yrs)	41.13	34.84	35.71	39.40	44.32	53.74	56.38	59.31	63.44	73.88	51.63

## Discussion

CPATs are rare cranial nerve tumors, the most common of which is VS. To date, only a few epidemiological studies have been published on tumors in this anatomical localization, and they are usually limited to VS only [8, 9, 17–23]. The present study included 6,173 patients diagnosed between 2011 and 2020 in Poland. The average incidence (1.99 / 100,000) is slightly higher than most results from the US—1.09 according to Kshettry et al. [17]; 1.11 according to Carlson et al. [18], 1.14 according to Cioffi et al. [19] and 1.2 according to Babu et al. [9]. On the other hand, a study by Marinelli et al. [20] showed an average incidence from 2006–2016 of 4.2 / 100,000, which is more than twice as high as in Poland. Canadian data shows average incidence of VS 1.27/100,000 [21]. The Stepanidis et al. study found an incidence of 2.21/100,000 in Denmark between 2003 and 2012 [22]. Other data from the Nordic countries from 1987–2007 indicate an incidence ranging from 0.61 among Finland men to 1.16 for Danish residents [23]. Higher results apply to Asian countries, in Taiwan 2.66 per 100,000 [8]. An increase in incidence was observed almost throughout the observation period. Some other studies also indicate a gradual increase in incidence over the years [20, 22]. This increase can be explained by better accessibility to diagnostics, greater details of imaging studies, and increased awareness among patients and physicians. The main impact is in our opinion mainly due to the availability of head MRIs. According to the "Maps of Health Needs" report from Ministry of Health [24], there were 168 MRIs in 2014, 339 in 2017 and 475 in 2020. We did not find environmental or other factors that may have increased the incidence of CPATs. The observed decline in the number of patients with CPATs in 2020 is likely due to the COVID-19 pandemic, that has reduced the number of health services provided, including diagnostic examinations.

The demographic factors we have shown are consistent with the literature. A slight preponderance of female patients was also found in US (51.98—53.59%) [9, 19, 20] and European studies (50.05–54.15%) [23]. The average age of patients was 53.78 ± 14.1 years, but most patients were diagnosed in the 60–69 age group. The higher incidence of VS was also observed in the age group in Taiwan patients [8]. In the U.S. studies, the incidence of VS was highest among adults aged 65–74 [17, 19], however, in another U.S. study by Babu et al. the average age of patients was 55 [9]. In a study of patients from Norwegian countries, the highest incidence rates were found in the age groups 45–54 and 55–64 years [23]. Stepadinis et al. showed a gradual increase in the mean age of patients from 48.2 years in 1976 to 57.1 years in 2011 [22]. The analysis of the patients' place of residence did not change over the analyzed period and roughly corresponds to the distribution of the Polish population into urban and rural areas. Territorial analysis conducted by Stepanidis et al. in Denmark also have shown no effect of place of residence on the clinical characteristics of CPATs [22].

Symptomatics of CPATs include many symptoms, with the most common complaints of patients being dizziness/vertigo, hearing impairment, tinnitus, headaches, sudden sensorineural hearing loss (SSNHL) and facial or trigeminal nerve dysfunction [25–27]. We selected a few of the above symptoms that are most frequently mentioned in the literature which may be directly defined using ICD-10 codes. The results confirm the widespread occurrence of these symptoms and vertigo has been the most common (43.48%). Other studies reporting symptoms of CPATs, particularly VS, are based on small groups of patients, making the results significantly different. In addition, underestimation of diagnoses may be a limitation, as described below. Thapa et al. reported hearing loss as main symptom in 72% of VS cases. Less frequently, patients complained of tinnitus (51%), dizziness (21%) and headache (14%) [25]. Smith et al. outlines that 90.7% of VS patients suffered from hearing loss, followed by dizziness/vertigo (61.2%), tinnitus (56.4%), and lateralized headache (9.3%) [26]. In particular, the presence of several of the above symptoms should be an indication to deepen the diagnosis to exclude CPAT.

SSNHL as a symptom of CPAT deserves additional attention. SSNHL is present in 1.12–20% of patients with CPATs [25, 28–32], but most of the mentioned studies were based on a retrospective single-center analysis. A systematic review conducted by Sweeney et al. [28] indicated that 7.9% of patients with VS experienced SSNHL before diagnosis. In our study, an average of 4.65% patients with CPATs experienced SSNHL. Although relatively rare, the diagnosis indicates a relatively high risk of CPAT, with an average of 6 cases per 1,000 people with SSNHL.

The management of VS include few therapeutic strategies including observation, microsurgical excision and radiosurgery (gamma-knife). There are many factors that may influence the choice of strategy. Patient related factors such as age, functional status, size and location of tumor and at last patient preference may change the course of management. Moreover, institutional and physicians biases may alter the treatment [9].

Several authors are rather consistent regarding the management of large VS with mass effect (Koos III or IV) and suggest microsurgical resection [33–35]. There is an ongoing debate in the literature in the topic of optimal pathway for management of small-to-medium size VS regarding functional outcomes.

In our study we have observed the constant increase of radiosurgery applied to patients with suspected VS and decrease the number of surgical interventions. It is probably associated with the introduction of gamma-knife in Poland in 2010. As gamma-knife became available for Polish patients and it has been proven in the literature that functional outcome regarding facial nerve and hearing preservation [27, 36] is promising more patients were referred for this treatment. The drastic decrease in surgeries performed in 2020 may also be associated with COVID-19 pandemic when surgeries other than life-saving were postponed.

Although, currently there are no research data suggesting that radiosurgery is superior to microsurgery or vice versa. It seems that tumor control is better after microsurgery than radiosurgery [37–39]. When it comes to hearing preservation, the main factor is hearing status before treatment and tumor size rather than the way of treatment [37–39]. The rates of hearing preservation is comparable for radiosurgery and microsurgery in small VS and ranges from 39 to 79% [39]. Hearing preservation and facial nerve impairment after microsurgical treatment is also dependent on experience and skills of the surgical team [38, 40] while progress in these outcomes with radiosurgery was done with lowering the Gy dosage applied to the tumor [37].

### Limitations

A limitation of the study is the lack of clinical data on individual patients. The retrospective study is based on data reported to the NHF, and definitions were based on the ICD-10 classification. The authors have no influence on the reliability of the reported data and the NHF database does not include histopathological diagnoses. But as described in "Materials and Methods," the defined study group includes all patients with CPATs, and among surgically treated patients, only patients with VS. Other treatments do not allow a certain histopathological identification. Due to the lack of a clear histopathological diagnosis in the NHF database, the abbreviation CPAT was used, but it should be noted that the selected cohort includes almost exclusively patients with VS. Therefore, the studies cited in the "Discussion" are often based on patients with VS and the results were compared to them. Also, basing on the data used in our study we could not discover and follow the patients initially directed towards observation and under observation until now.

The study certainly has the advantage of being a cohort study covering the entire population of the country and a 10-year period. It is also the first study of patients with CPATs covering the Polish population.

## Conclusions

To the best of our knowledge, this is the first nation-wide study aimed at searching the incidence of CPAT in Poland. The total incidence of CPAT and demographic characteristics of patients were comparable to other studies. Our study demonstrated that over the past decade increasingly more patients are being treated with gamma knife and fewer with microsurgery. SSNHL is an uncommon manifestation of CPAT, but proper diagnosis should be undertaken in this group of patients because the risk of VS diagnosis is greatest.

### Supplementary Information

**Additional file 1.** 

## Data Availability

Please contact authors for data requests (Michał Żurek MD—email: michal.zurek@wum.edu.pl). The data belong to Department of Analyses and Strategies, Ministry of Health and are not available to share unless in the form included in the manuscript.

## Abbreviations

VS: Vestibular schwannoma
CPAT: Cerebellopontine angle tumor
IAM: Internal acoustic meatus
NHF: National Health Fund
SSNHL: Sudden sensorineural hearing loss
