# Supplementary material for: Nationwide clinico-epidemiological treatment analysis of adult patients with tumors of cerebellopontine angle and internal acoustic meatus in Poland during 2011–2020
Source: BMC Public Health. 2023 Sep 6;23:1735. doi: 10.1186/s12889-023-16551-5 (PMC10481480; doi:10.1186/s12889-023-16551-5)
Supplement: Supplementary file 1 — Additional file 1. [file 12889_2023_16551_MOESM1_ESM.docx]

Supplementary Material 1. ICD-9 and ICD-10 code descriptions used in the study.

| ICD-9 codes | Description |
| --- | --- |
| 01 | Incision and excision of the skull, brain and meninges |
| 04.011 | Excision of a neuroma of the acoustic nerve |
| 04.012 | Excision of a neuroma of the acoustic nerve with craniotomy |
| 92.27 | Stereotactic teleradiotherapy with gamma rays from multiple microsources |
| 92.3 | Stereotactic radiosurgery |
| 92.2 | Radiotherapy and nuclear medicine |
| ICD-10 codes |  |
| D33.3 | Benign neoplasm: cranial nerves |
| H91.2 | Sudden idiopathic hearing loss. |
| H90.3 | Sensorineural hearing loss, bilateral |
| H90.4 | Sensorineural hearing loss, unilateral with unrestricted hearing on the contralateral side |
| H90.5 | Sensorineural hearing loss, unspecified |
| H90.6 | Mixed conductive and sensorineural hearing loss, bilateral |
| H90.7 | Mixed conductive and sensorineural hearing loss, unilateral with unrestricted hearing on the contralateral side |
| H90.8 | Mixed conductive and sensorineural hearing loss, unspecified |
| H93.1 | Tinnitus |
| R51 | Headache |
| R42 | Dizziness and giddiness |
| H81 | Disorders of vestibular function |
